# A Rare Case of Demodicosis Following Treatment With Oral Fluconazole

**DOI:** 10.7759/cureus.33309

**Published:** 2023-01-03

**Authors:** Joshua A Bezecny, Emily Bolton, Matthew H Taylor, Elizabeth G Berry

**Affiliations:** 1 Dermatology, HCA HealthONE, Denver, USA; 2 Dermatology, Oregon Health & Science University, Portland, USA; 3 Medical Oncology, Providence Cancer Institute Franz Clinic, Portland, USA

**Keywords:** fungal microbiome, fluconazole, permethrin, ivermectin, antifungal, blepharitis, demodicosis, demodex

## Abstract

*Demodex folliculorum* and *Demodex brevis* are commensal human ectoparasites that reside within or near hair follicles and have been highly associated with rosacea-like papulopustular skin eruptions. We present an interesting case of recurrent, iatrogenic demodicosis in a 56-year-old man. We suspect this to have been triggered by antifungal therapy given it occurred twice closely following azole treatment. We propose that oral antifungals in the setting of immunosuppression can alter the skin microbiome, facilitating *Demodex* proliferation.

## Introduction

*Demodex folliculorum* and *Demodex brevis* are commensal human ectoparasites that reside within or near hair follicles. Numerous studies have found significantly higher populations of inflammatory cells in skin biopsies of patients with rosacea-like papulopustular skin eruptions containing high *Demodex *counts [1]. Blepharitis and meibomian gland dysfunction have also been associated with demodicosis [2]. More extensive inflammation with a more diffuse facial and truncal distribution is seen with demodicosis secondary to immunosuppression [3].

Demodicosis is known to occur in the context of immunosuppression [4,5]. Demodicosis following oral antifungals is a unique association not previously reported. Here, we present a case of demodicosis following treatment with fluconazole.

## Case presentation

In 2019, a 56-year-old man with a history of metastatic melanoma, treated most recently with nivolumab complicated by pneumonitis requiring high-dose prednisone and infliximab, developed a pruritic pustular eruption on the scalp, face, chest, and back. He also developed a sensation of eye grittiness and photophobia. He had started nivolumab for metastatic melanoma in the fall of 2018, and prednisone 140 mg daily (2 mg/kg/day) was started three months later for treatment of immune-mediated pneumonitis. One month prior to the onset of the rash, he received one dose of infliximab 10 mg/kg, which he also had in 2016 for colitis secondary to adjuvant immunotherapy with ipilimumab. Additionally, he recently completed a two-week course of fluconazole and several doses of itraconazole for prednisone-induced thrush.

**Figure 1:**
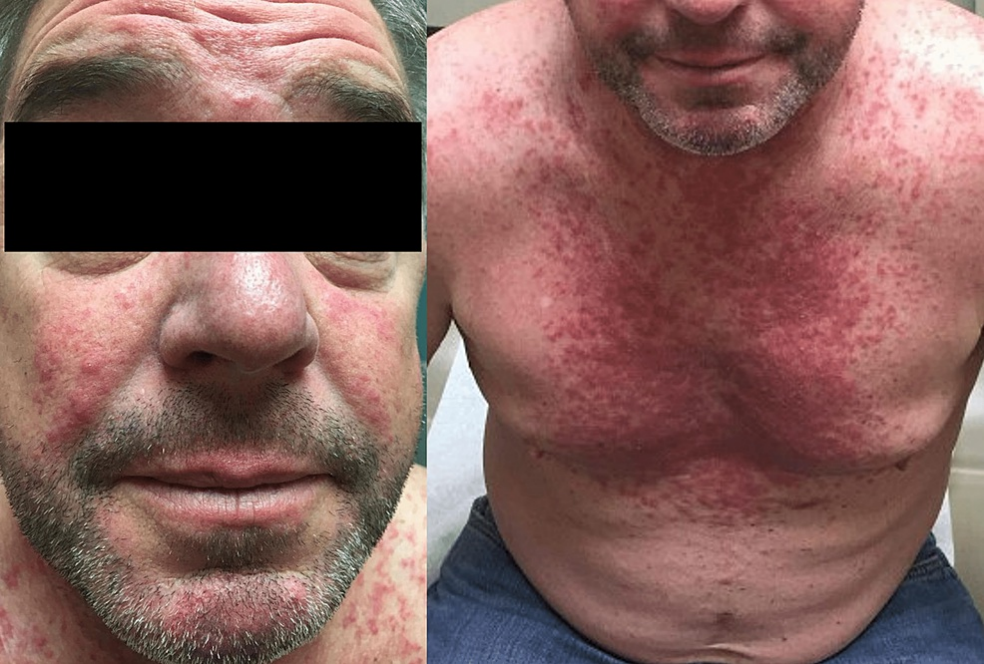
Papules and pustules on the face and chest, respectively.

**Figure 2:**
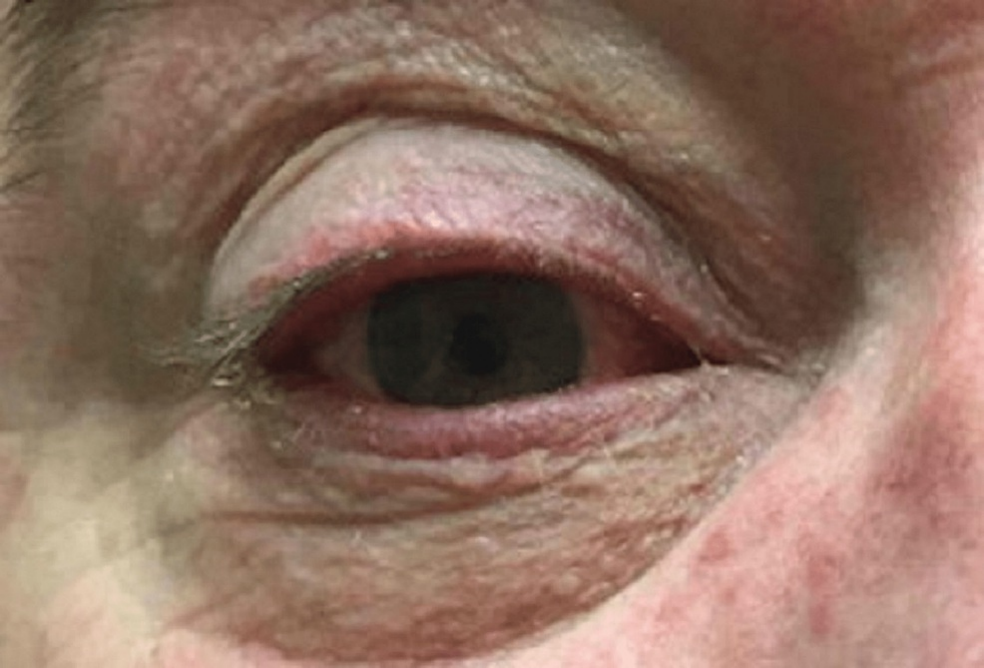
Eyelid margin erythema and conjunctival injection.

He presented to the emergency department and received antihistamines and topical steroids. Two days later, he presented to the dermatology clinic with a worsening pruritic eruption. On examination, he had diffuse, erythematous papules and pustules covering his face, neck, chest, and back (Figure 1). He had bilateral eyelid margin erythema and conjunctival injection (Figure 2). Differential diagnoses included demodicosis, bacterial folliculitis, pityrosporum folliculitis, eosinophilic pustular folliculitis, acne, and rosacea. He was started on oral doxycycline 100 mg twice daily, clobetasol 0.05% scalp solution, triamcinolone 0.1% cream for the trunk, and hydrocortisone 2.5% topical cream for the face. The bacterial culture of pustules was negative. A punch biopsy of the left chest showed suppurative folliculitis. Ophthalmology saw him urgently due to the photophobia and diagnosed him with meibomian gland dysfunction. He was started on neomycin/polymyxin B/dexamethasone ophthalmic ointment and artificial tears.

**Figure 3:**
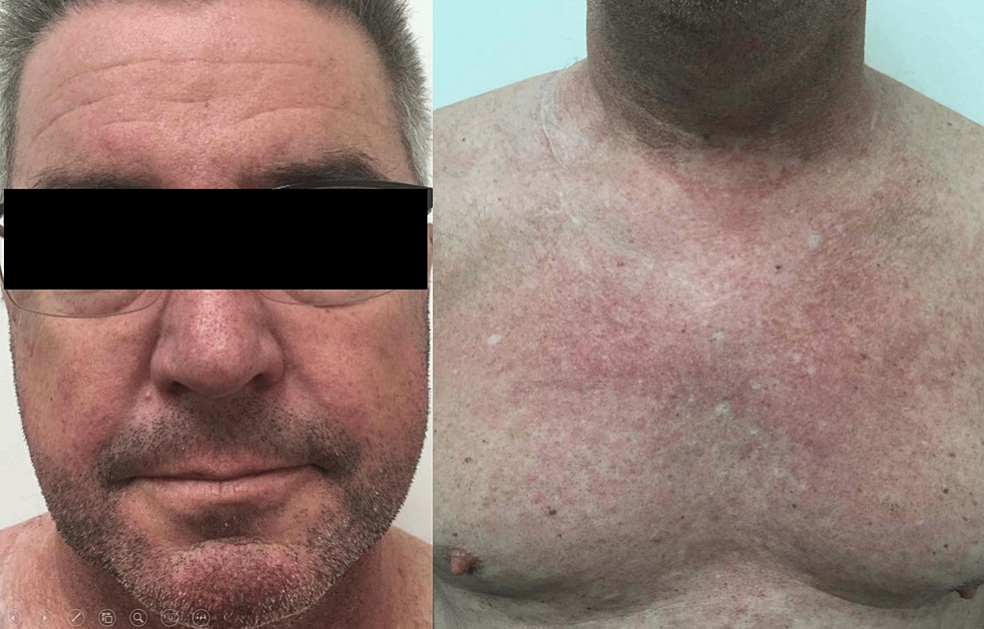
Resolution of facial skin eruption and significant improvement of his chest, respectively.

One week later, he returned without improvement. Topical steroids were discontinued. Microscopic examination of several eyelashes and the contents of pustules did not show mites, but empiric treatment for suspected *Demodex *was started, including two doses of oral ivermectin (15 mg, one week apart) and permethrin 5% cream twice daily. Ketoconazole 2% shampoo three times weekly was also started as empiric treatment for pityrosporum folliculitis. He continued doxycycline 100 mg twice daily. Two weeks after the onset of the rash, he returned with marked improvement in his symptoms. Doxycycline was reduced to 100 mg daily for 10 days and ketoconazole shampoo was discontinued. He was instructed to continue permethrin cream twice daily until the resolution of symptoms. One month after the onset of the rash, he returned to the dermatology clinic with a resolution of his facial skin eruption and significant improvement in his back and chest (Figure 3).

After several months, he returned to the clinic with a similar pustular eruption of the scalp, face, chest, and back. He was no longer on oral prednisone or infliximab infusions, and nivolumab had not been restarted. Due to persistent oral candidiasis from his high-dose steroids, he had been prescribed oral fluconazole. After the first dose of fluconazole, he developed a pruritic papulopustular rash in the distribution described previously. No mites were identified from scrapings of pustules and eyelashes. However, given his consistent morphology and prior improvement on empiric treatment for demodicosis, he was again treated with the same regimen of oral ivermectin and topical permethrin. The eruption cleared within a few days and has not returned. *Demodex* mites were not isolated, but prior studies have shown that biopsy can have false-negative results, even with very high mite density [6,7].

## Discussion

Demodicosis is associated with immunosuppressive medications (mycophenolate, azathioprine, tacrolimus, prednisone, and chemotherapeutic agents) and systemic illnesses (human immunodeficiency virus infection, leukemia, lymphoma) [4,5]. The immunocompromised state can result in the disruption of innate skin immunity, promoting increased colonization and population expansion of *Demodex *mites [4,5]. Our patient’s immunocompromised state (secondary to high-dose prednisone and infliximab) likely predisposed him to *Demodex *proliferation. There has been one report of demodicosis from infliximab [8]. We feel that it is unlikely that nivolumab was associated with this skin eruption. There have not been reports of nivolumab-associated demodicosis, and our patient had not received this medication for several months.

The temporal association of oral fluconazole with our patient’s eruption is interesting. This raises the question of whether antifungal exposure had caused a fungal microbiome shift, further facilitating *Demodex *mite proliferation. Fluconazole and amphotericin B have been shown to alter the gut fungal microbiome in mice [9]. Diverse fungal communities exist at all barrier surfaces, and disruptions can negatively affect the host immune response [10]. The possible association of antifungal treatment, alteration in commensal skin fungi, and demodicosis merits further thought and investigation in future studies. We are not aware of prior studies associating oral antifungals with demodicosis.

## Conclusions

Our patient presented with an eruption clinically suspicious for demodicosis initially in the setting of immunosuppression and antifungals for oral candidiasis, which recurred months later with a single dose of fluconazole in the absence of other immunosuppressive agents. Demodicosis should be considered in patients with pruritic papulopustular, rosacea-like eruptions in the setting of immunosuppression. Our case also demonstrates that oral antifungal agents may play a role in disrupting the skin microbiome to facilitate *Demodex *proliferation. The premise of antifungal treatment disrupting natural flora and facilitating demodicosis merits further study.
