# Correction: A Rare Case of Demodicosis Following Treatment With Oral Fluconazole

**DOI:** 10.7759/cureus.c93

**Published:** 2023-01-11

**Authors:** Joshua A Bezecny, Emily Bolton, Matthew H Taylor, Elizabeth G Berry

**Affiliations:** 1 Dermatology, HCA HealthONE, Denver, USA; 2 Dermatology, Oregon Health & Science University, Portland, USA; 3 Medical Oncology, Providence Cancer Institute Franz Clinic, Portland, USA

The author order has been changed at the request of the authors: Matthew H. Taylor and Elizabeth G. Berry have switched positions and are now the third and fourth authors, respectively. The authors deeply regret that this error was not identified and corrected prior to publication.

